# Facilitating Emergency Remote K-12 Teaching in Computing-Enhanced Virtual Learning Environments During COVID-19 Pandemic - Blessing or Curse?

**DOI:** 10.1177/0735633121992781

**Published:** 2021-12

**Authors:** Tamar Shamir-Inbal, Ina Blau

**Affiliations:** 1 Department of Education and Psychology, The Open University of Israel, Ra'anana, Israel

**Keywords:** emergency remote teaching – ERT, distance learning, K-12 in COVID-19 pandemic, teachers, pedagogical strategies, elementary and secondary schools

## Abstract

This study explored teacher experience in leading Emergency Remote Teaching (ERT) in K-12 and conducting blended synchronous and asynchronous instruction during the COVID-19 pandemic. The study’s purpose was to understand the pedagogical, technological, and organizational challenges and benefits of computing-enhanced digital learning environments, and to explore teachers' pedagogical strategies. This study employed a qualitative research paradigm using nation-wide, online samples, which included 133 elementary and secondary school teachers from Hebrew-speaking and Arabic-speaking schools in Israel. Participants were asked to share their perspectives and experiences of ERT through open-ended questions in an online questionnaire. The bottom-up analysis of the data, based on the Grounded Theory approach, yielded 1,822 statements reflecting teachers' perceptions of pedagogical, technological, and organizational challenges (N = 580) and benefits of ERT (N = 827). The analysis also revealed a variety of pedagogical distance learning strategies used by teachers (N = 415). The study raises the need to turn a curse into a blessing by incorporating the experience of remote technology-enhanced learning and online activities into the school agenda on a regular basis. Thus, teachers and students would develop important digital competencies and be prepared for the next emergency event. The implications of our findings for educational theory and practice of educational computing are discussed.

## Introduction

Maintaining learning in a time of global disruption in order to support students' well-being has become a major challenge for the entire global education community (Huang et al., 2020). Due to emergencies and traumatic events, such as the COVID-19 pandemic, schools are unable to carry out their activities normally. At such times, education systems should provide Emergency Remote Teaching (ERT) as an alternative way of preserving the teaching-learning processes (Hodges et al., 2020; Laprairie & Hinson, 2006). ERT is conducted in synchronous or asynchronous environments using various devices with internet access.

Distance Learning (DL), which is the base of ERT, can enable education anywhere and anytime, and during an emergency event it can create a structured daily routine with meaningful and creative activities for students (Burde et al., 2017). As a result of the COVID-19 pandemic, Ministries of Education (MoE) around the world have been forced to conduct schooling for students in their homes. Online learning has ensured that, despite the lockdown and inability to attend schools (Kong, 2020), education can continue with a minimum disruption of the routine learning process until it is safe again to return to face-to-face learning (Xie & Yang, 2020).

However, effective online learning during emergency events is based on the relevant skills that teachers and students have developed during regular learning. Unfortunately, evidence provided by the OECD’s Programmed in International Student Assessment (PISA) shows that most of the education systems that participated in PISA in 2018 did not offer opportunities to teach online (Sälzer & Roczen, 2018). One of the basic barriers was a lack of adequate infrastructure, since online learning requires a computer with internet connection in order to complete learning assignments at home. Other parts of PISA explored how well education institutions were equipped with appropriate technology and to what extent teachers were prepared to engage their students in online learning (Sälzer & Roczen, 2018). Thus, not surprisingly, the COVID-19 pandemic has raised a variety of challenges for which the education systems were not prepared (Kong, 2020). The change to ERT was especially challenging because of traditional pedagogy based on teachers transferring information and students absorbing this content (Cheng, 2020).

Although distance learning has been studied in depth, research of distance learning in the K-12 setting is still very limited. (Harris-Packer & Ségol, 2015; Schwartz et al., 2020). The purpose of this study was to explore the challenges that teachers face in implementing online distance learning processes in times of crisis. This was in order to understand the solutions they have adopted, and the added value, if any, of such learning activities in order to suggest appropriate pedagogy for future emergencies and restricted mobility events.

## Literature Review

As the COVID-19 pandemic has spread throughout the world, the question of how to continue schooling has become a major challenge in most global education systems (Kong, 2020). Moving instruction to Emergency Remote Teaching (ERT) is based on the assumption that distance learning can be an effective, supportive routine during the time of disruption. ERT is expected to occur immediately and to enable flexibility in teaching and learning anywhere and anytime (Cheng, 2020; Hodges et al., 2020). It therefore provides students with increased choices about where, when, and how learning will occur (Cheng, 2020). Accordingly, distance learning (DL) focuses on web-based teaching, learning, and instructional design in synchronous and asynchronous environments and raises a variety of new requirements related to technology operation, teaching skills, and management (Zhang, 2020).

DL can be described by four characteristics (Simonson et al., 2011). First, DL is not self-paced study and is obtained from the agencies that conduct traditional face-to-face education. Second, geographic separation is inherent in DL, and time might also separate students and teachers. Third, interactive communication connects the learning groups with each other and with the teacher. The connection of learners, teachers, and instructional resources becomes less dependent on physical proximity as digital platforms and communication tools become available, and this contributes to the rapid expansion of DL. Finally, DL, like any education process, establishes a learning triangle composed of students, a teacher, and instructional resources.

### Opportunities and Challenges of Distance Learning in K-12

Education leaders in K-12 embrace online distance **learning opportunities** for several reasons. The benefits of online DL can lead to changes in the nature of education, motivate students to participate in online learning activities, expand educational access, and encourage students to function as self-regulated and independent learners (Blau & Shamir-Inbal, 2017a; Harris-Packer & Ségol, 2015). It provides students with a variety of choices, convenience to their needs, and personalization that supports effective teaching and learning processes (Engelbertink et al., 2020). Moreover, technology can promote the construction of new knowledge based on a variety of pedagogical approaches and a wide range of learning resources (Blau & Shamir-Inbal, 2017b; Blau, Shamir-Inbal, & Avdiel, 2020). This can promote development of social and collaborative skills, as well as personal relationships among participants (Blau, Shamir-Inbal & Hadad, 2020; Huang et al., 2020).

However, the need to move rapidly to an online mode raises various **challenges** for school administrations, teachers, and students (Rice, 2006). *The challenges for school administrations* due to the COVID-19 pandemic included controlling logistical issues, such as handling the relationship between the school's unified regulations and the autonomous arrangements of teachers and students (Dong, 2020; Schwartz et al., 2020). *The challenges for teachers* were caused by insufficient technological and pedagogical support or by inexperience in using online tools on a daily basis. Teacher guidance and support are essential in order to develop students' abilities to navigate on their own in the online learning world (Dong, 2020). In order to guide students effectively through learning activities at home, teachers had to improve their own skills of integrating online resources and digital tasks into their practices and increase communication with students and their parents (Kong, 2020). Moreover, parental involvement became a key component in ERT. Accordingly, in order to maximize parental support and cooperation, teachers had to establish effective teacher-student and teacher-parent e-communication through school platforms or social networks (Blau & Hameiri, 2017; Cheng, 2020; Hadad, Shamir-Inbal, & Blau, 2020). *The challenges for students* included improving their self-regulated learning skills, enhancing their interest and responsibility during the home learning process, and making appropriate adjustments in their learning routines.

Based on COVID-19 experience in China, Huang et al. (2020) presented suggestions of organizing ERT to **overcome various challenges**. These included (a) providing facilities necessary for digital learning operation and infrastructure; for example, supplying access to e-learning resources and education programs, (b) e-learning training for teachers which facilitated adaptation of appropriate pedagogy for such settings, (c) strengthening internal leadership and teacher-to-teacher learning and cooperation, and (d) collaboration between several sectors (governmental, telecommunication, educational enterprise, etc.).

**Figure 1.**
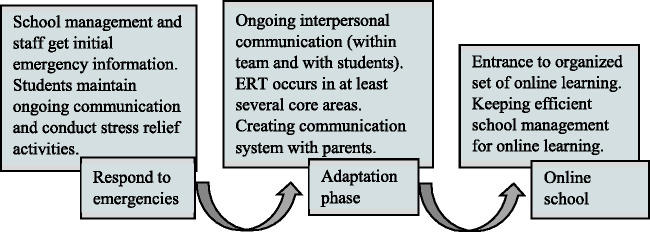
Instructions to Assist Organizing Online Learning During Emergency Time.

Consistent with Chinese experience, in order to assist school organization during the COVID-19 pandemic, the Israeli Ministry of Education (MoE, 2020) published a set of instructions for all schools and for all age groups. On *the national level,* ERT was promoted by recording and broadcasting training sessions for teachers and lessons for students. Further, high-quality learning activities in various learning topics were uploaded to a national pool of activities for the benefit of teachers across the country. At the *teacher level*, teachers were required to conduct ERT from their homes, prepare learning activities, and send assignments to their students through a Content Learning Management System platform (CLMS). In addition, school staff were guided to provide real-time communication in the form of whole class *Zoom* (https://zoom.us/) sessions, to maintain continuous contact and social interactions, and to provide emotional support. At the *student level*, ERT was conducted synchronously and asynchronously at home. In most cases, it was recommended that students be assisted by parents or other family members in technical issues and to complete learning tasks. Figure 1 describes the set of instructions for organization regarding ERT, as planned and published by the MoE.

### Synchronous, Asynchronous, and Blended Distance Learning

According to this model, the guidelines (Israeli MoE, 2020) suggest applying DL that combines synchronous and asynchronous modes. ***Synchronous sessions*** enable social activities in place of the usual meetings in class. Likewise, similar to face-to-face classes, synchronous learning sessions simulate regular class lessons based on whole class activities involving students in discussions, individual writing, or peer feedback. Using videoconferencing platforms, such as *Zoom,* for interactive synchronous online sessions, enables two-way communication through watching (digital camera), speaking and listening (microphone and headphones), and sharing screens for presentation and teamwork (Blau et al., 2017; Weiser et al., 2018). Note that the popularity of *Zoom* as a tool for DL sessions ignores the fact that it can lead to traditional teacher-centered pedagogical models (Blau et al., 2017). Student-centered synchronous videoconferencing can also promote discussions of learning topics, student presentations of their learning artifacts, and peer feedback (Blau & Shamir-Inbal, 2017b; Weiser et al., 2018).

***Asynchronous tasks*** aim to promote students' independent learning. In asynchronous activities based on a platform, for example, in *Google G-Suit for Education* (https://edu.google.com/products/gsuite-for-education/), the learning topic can be uniform or differentiated and can be studied individually or collaboratively in small groups (Sharma & Kumar, 2017). Such processes can lead to various learning outcomes, including designing digital artifacts by students (Blau & Shamir-Inbal, 2017b). The teacher's role in asynchronous activities is to offer guidance and scaffold independent learning of students.

The combination of synchronous and asynchronous online learning - ***blended learning*** - can be an effective way to engage students as active participants in the learning process (Allen & Seaman, 2017; Blau & Shamir-Inbal, 2017b; Galley et al., 2010). This combination can assist the continuity of schooling during ERT by maintaining the teaching and learning routine and can help teachers monitor the well-being of their students.

### Research Goals and Questions

This study explored the different aspects of challenges and benefits in conducting online distance learning processes in times of crisis, and the pedagogical strategies teachers used during ERT. Accordingly, the research questions were:

1. What are the main challenges and benefits of emergency remote teaching during COVID-19 in pedagogical, technological, and organizational aspects?
2. What are the pedagogical strategies teachers employ for ERT?

## Method

To address the research questions, we employed qualitative research methods that enabled us to understand teachers' perspectives and their actual pedagogical strategies in ERT during the COVID-19 pandemic.

### Participants

The online sample consisted of 133 educators from all the districts of the Israeli MoE. The educators were 54 (40.5%) homeroom teachers, 44 (33.1%) subject-matter teachers, 22 (16.5%) school or regional ICT coordinators, and the rest were school principals or vice principals. Among the participants, 69 (51.9%) worked in elementary schools (grades 1–6) and the rest in secondary schools (grades 7–12). The vast majority of the participants were from Hebrew-speaking schools, while 8.3% were from Arabic-speaking schools. Most of the sample (98, 73.7%) consisted of educators with seniority of more than 10 years, while 20 (15%) had seniority of 6–10 years and 15 (11.3%) up to 5 years. Out of the participants, 44 (43.2%) also had experience in teacher training in addition to teaching.

### Instruments and Procedure

The data was collected in May 2020 after the first few months of the COVID-19 pandemic and following ERT experience in the entire education system. The study received approval from the Institutional Ethics Committee. Teachers were asked to share their perspectives and pedagogical practices of ERT through an online questionnaire distributed through teacher groups on the Facebook social network. The questionnaire included a multiple-choice question in which participants were asked to report whether their ERT was performed mainly in a synchronous, asynchronous, or a blended mode combining both. Other questions were open-ended in order to gain an in depth understanding of the teachers' perspectives and practices regarding this experience. Examples of the questions were as follows: Please describe your online teaching experience during COVID-19. How was the school organized to assist in conducting online distance learning? Whether and in what way was the school prepared in advance for such instruction? What immediate staff training was offered, if any? What were the key challenges you faced, and how did you deal with them? Please describe in detail two learning activities that you conducted with your students during COVID-19. Describe which students performed these activities and what feedback, if any, you received from their parents about your teaching and/or the students' learning experience. What has changed, if so, in your attitude towards technological and/or pedagogical aspects of online distance learning as a result of ERT during COVID-19?

A thematic analysis of the participants' answers was conducted on the open-ended questions. The coding was not exclusive; namely, each statement could be attributed to several categories. To ensure inter-rater reliability, 25% of the statements were analyzed by a second rater and the agreement level was high, Cohen's kappa = .86.

**Figure 2.**
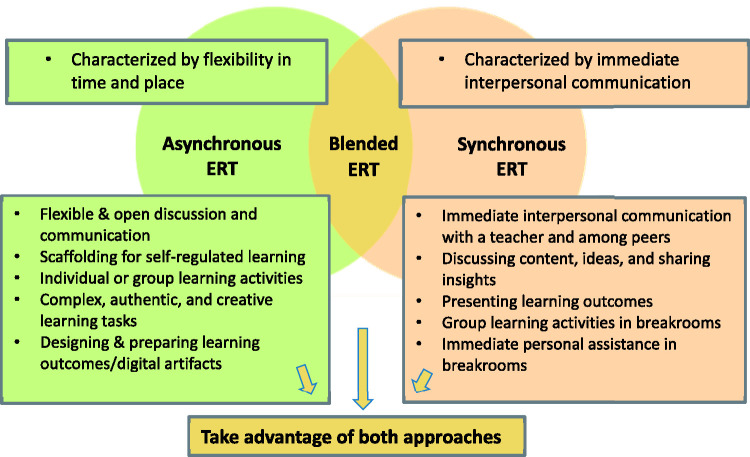
Teaching Strategies and Characteristics of Emergency Remote Teaching.

This analysis employed a qualitative methodology in accordance with the principles of the Grounded Theory approach (Corbin & Strauss, 2014), which extracted data from the participants' narratives. This methodology drew on the participants' descriptions of their experiences and their interpretation of these experiences, which could enable researchers to understand phenomena in the context in which they occurred. In grounded theory analysis coding is performed on three levels (Berthelsen et al., 2018; Corbin & Strauss, 2014): 1) Initial and open coding in the inductive phase. The bottom-up analysis of the answers yielded 1,822 statements which were categorized using a thematic analysis technique. These statements were coded and grouped into three major categories: teachers' challenges (N = 580), teachers' benefits (N = 827), and ERT strategies used by teachers (N = 415). 2) The more focused and selective coding according to concurrent concepts and categories in the deductive phase. This analysis, among others, grouped several types of teachers' professional challenges – pedagogical, technological, and organizational, as well as teachers' personal challenges as a result of ERT. 3) Theoretical coding to structure the theory to a progressive level of abstraction. This analysis enabled us to structure pedagogical strategies found in this study into a visual representation of teaching strategies and characteristics of ERT (Figure 2).

## Results

### Challenges and Benefits of ERT

Tables 1 and 2 describe the challenges and benefits of teachers and students in the context of distance learning during the COVID-19 event. Note that the challenges of students in Table 1 present statements based on teachers' perspectives.

**Table 1.** Challenges Teachers and Students Faced During ERT (N = 580).

Main category	N, %	Representative statements
Challenges of teachers (N = 269 statements)		
*Pedagogical challenges-* Difficulties in conducting differential learning, assessing student performance, or maintaining contact with students	80, 30%	“It was challenging to learn by myself how to teach differently from how I usually teach.” (T116) “In distance learning I had difficulty opening up to this new way of teaching.” (T9)
*Technological challenges-* Lack of technological knowledge or suitable equipment	50, 19%	“I had difficulty learning new platforms … Constant trial and error was very tiring.” (T39) “The need to use various computer programs that I wasn’t familiar with was stressful.” (T58)
*Organizational and systemic challenges-* Lack of readiness for ERT Lack of organizational guidance or support	93, 34%	“At first, the situation [with K-12 during the pandemic] was uncertain. It was difficult to prepare ourselves when it was unclear what to do and how.” (T12) “The guiding protocol from the MoE confused everybody. Each teacher acted as he/she found appropriate. There was no guiding hand … I planned to hold classes with lectures that were broadcast on the national level, but due to technical problems they were canceled 90 minutes before the scheduled time.” (T44)
*Personal challenges and overload-* Overload caused by preparation of new tasks, long work hours at home spread over the day and combined with family care	46, 17%	“I was required to learn new [things] … in uncomfortable circumstances and pressured by time … My main challenge was to teach from home when my own kids were around.” (T52) “… It was difficult to teach in an online mode because my kids needed to use the one computer we have at home.” (T5)
Challenges of student**s** (N = 311 statements)		
The challenge for students to become independent learners	138, 40%	“The remote independent learning was not easy. The students are familiar with learning in the classroom with the possibility of asking questions and receiving immediate explanations. They struggled in studying alone.” (T131) “For many students distance and independent learning is not appropriate. They cannot learn alone, they do not understand what to do, and they need the teacher’s assistance.” (T119)
The challenge of dealing with students' loneliness and anxiety	21, 7%	“Students had emotional difficulties in dealing with the situation, fear of the unknown, and longing for the familiar routine, for their friends and teachers.” (T120) “Most of them missed friends. Some were bored and some complained about quarrels with their siblings.” (T117)
Student technologicalchallenges – skills of operating devices and applications, lack of equipment	99, 32%	“Distance learning requires that each student has a computer. There are many students with low socio-economic status who do not have a computer [for every child in the family], or their computer is broken or not connected to the internet because of the limited family budget.” (T6) “The learners had a difficult time. The majority of them have no computers at home, so they worked on their mobile phones, which did not enable them to open large documents or write in them.” (T112)
The challenge that resulted from a lack of parental support	53, 17%	“Some parents could not assist their children because they did not know how to help them or because they had no time for it.” (T78) “Some parents let the children stay in bed instead of waking them up to learn, just so they would not have to deal with them.” (T20)

**Table 2.** Benefits of Teachers During Emergency Remote Teaching (N = 827).

Main category	N, %	Representative statements
*Pedagogical aspects* **-**Experiencing new ways and tools for teaching	89, 11%	“I felt significant personal development as a result of designing new learning tasks and innovative teaching materials. For me, it was an extensive training, a period of learning and professional growth.” (T97) “I repeated grammar rules we learned before the COVID-19 pandemic. I opened a collaborative document, referred my students to the guidance video that I prepared, and asked them to use words from a list in order to write the sentences with different tenses. Afterward they were asked to provide feedback to their peers. Finally, we checked the sentences together. It was a new experience teaching that way.” (T28)
Teachers as designers	25, 3%	“I gained a different kind of communication with students in order to give them differential tasks. This required my flexibility as a teacher in preparing a new learning design tailored to my students' needs.” (T104) “I design digital lessons using Nearped. I try to make these lessons interactive, thus the students need to be active participants in the learning process.” (T32)
*Technological aspects-* Awareness of new digital tools for teaching	79, 10%	“During ERT I learned to use a lot of digital tools that I did not need before. I figured out that this is not as scary as I assumed it would be.” (T18) “My experience in using ERT has been a real opportunity to use all the digital tools I have learned in recent years.” (T36)
*Personal aspects-* A sense of personal empowerment Developing the ability of self-directed learning	60, 7%	“I experienced amazing personal growth! … I faced difficulties but developed the ability to solve problems on my own.” (T81) “This was a golden opportunity … that forced me to become an independent learner, to seek new tools and develop new ideas….” (T20)
*Systemic aspects-the school level* School readiness for ERT	40, 5%	“Every year we participate in emergency distance learning exercises. As soon as it was decided to close schools, we were provided with instructions of what to do and how to do it.” (T5) “We received appropriate training in using digital tools before COVID-19. The school is innovative and leads toward technology integration. There is encouragement from the school principal, as well as from leading teachers, to integrate technology in our teaching process.” (T28)
Organizing for school internal, mutual support	121, 15%	“Zoom staff sessions were held weekly with personal guidance for those who needed it for phone calls and WhatsApp messages.” (T16) “We had synchronous team meetings to learn new ways of communicating, and teachers with experience and knowledge helped the others.” (T21)
Sharing content and learning activities with peers	124, 15%	“Teachers were committed to helping each other, sharing learning materials and useful sites, helping design learning activities, and more.” (T20) “Our staff shared learning materials with each other and shared content that was suitable for other teachers.” (T5)
Designing school plan and schedule	40, 5%	“The school principal, vice principal, and ICT coordinator met together and planned how to organize distance learning for the school.” (T103) “The pedagogical coordinators created a schedule, so that each teacher in the team knew what they were teaching and when; thus, it was well-organized and clear for the students as well.” (T29)
*Systemic aspects-the school community level - students* Teacher-student communication Taking care of student well-being	130, 15%	“As an educator, I opened a window to see my students' world and to get to know their hobbies and their pets. This straightened out connections between us.” (T41) “The students were very involved. They waited every morning for our meeting … we talked, we laughed, we performed fun tasks, and maintained a sense of daily routine.” (T87)
*Systemic aspects-: the school community level - parents* A sense of success based on parental involvement	107, 13%	“The parents were very involved, caring, and ready to assist. They supported their children and helped them. I received warm feedback and encouragement.” (T81) “The responses I received from parents were positive. They expressed satisfaction that a learning routine and continuity were maintained and connected the students.” (T82)
*Systemic aspects-the national level* Use of learning materials designed by digital content providers	12, 1%	“The assignments that we gave our students were taken from different educational content websites.” (T128) “There are so many wonderful online learning platforms in which every student can find content of his or her interest. I gave students tasks to perform in these educational platforms so that they could practice.” (T120)
Use of learning materials transmitted by the MoE		“The education channel transmitted daily lectures in all subject matter. taught They were taught by leading teachers chosen by the Ministry of Education and helped the students maintain their learning.” (T2) “The Ministry of Education presented interesting lessons for the students in a variety of subjects.” (T28)

As described in the table 1, participants presented various types of challenges which hampered distance learning. The pedagogical challenges reported by teachers were based on the need to adapt familiar teaching methods to the new medium, to the level of different students, and to the lack of face-to-face contact with them. In addition, teachers reported difficulties with the organizational aspect of establishing distance learning. These included a negative attitude and lack of support from the MoE, and/or difficulties with the school's internal organization, which needed to react quickly and differently from usual school functioning. Each year there is a national exercise which aims to prepare schools for functioning in an emergency situation. However, not all schools were ready for such an immediate change and therefore found it difficult to adjust to ERT.

There were also technological challenges that emerged from the need to start using ERT immediately and not always with sufficient training or various types of digital tools. Interestingly, the personal challenges that resulted from the necessity of teaching from home, rather than in school, were the lowest. Other challenges related to teachers’ emphasis on the difficulties of students to perform as independent learners, to develop self-regulated skills, as well as to collaborate and develop a study routine. They described difficulty in keeping constant contact with students who woke up late, did not join the class *Zoom* meetings, or disappeared from the screen. Therefore, interpersonal communication with them was lacking. Likewise, the teachers addressed challenges related to lack of support and assistance from parents. Interestingly, students' emotional distress was mentioned less as a relevant challenge to this period.

This table shows that, despite the challenges, most teachers held positive perceptions about their experience in distance learning and teaching during the COVID-19 emergency. Teachers perceived the experience as an opportunity for personal and professional empowerment that was forced upon them by the circumstances. Many of them reported a sense of success in dealing with this task. The participants reported that they acquired new teaching methods and were introduced to a range of innovative technological tools. They also experienced a sense of motivation, cooperation with students, and appreciation and support from the parents. According to the findings, most schools succeeded in getting organized quickly and moved immediately to ERT. An important benefit at the school level occurred when teaching staff were required to support each other, design and share learning materials and tasks, and divide responsibilities within their teams. Moreover, they were able to provide mutual support and create a suitable schedule for the ERT curriculum.

### Pedagogical Strategies for ERT

Experience in ERT required teachers to implement different, creative, and diverse teaching strategies. The findings revealed a variety of teaching, learning, and assessment strategies used by teachers (N = 335). Most of the teachers claimed that they preferred to use asynchronous channels (48, 36%) or blended learning (46, 35%), while fewer teachers reported that they employed mainly synchronous ERT (39, 29%). Table 3 introduces the range of strategies used by participants during their distance learning experience.

**Table 3.** Mapping Teaching Strategies Through Emergency Remote Teaching.

Main category	N, %	Representative statements
Mapping teaching strategies (N = 415 statements)		
Synchronous learning activities through videoconference sessions	68, 17%	“Lesson openings were synchronous via Zoom. I explained the task and answered students' questions. Then the students performed tasks, and at the end of the lesson we all went back to the meeting room and had a synchronous debate to conclude the lesson.” (T130) “At the beginning of the lesson I demonstrated an experiment in physics via Zoom, and then each student performed a similar experiment and sent a photo in order to report his experiment outcome.” (T120)
Gamification in videoconference class sessions	30, 8%	“Each lesson opened with a quiz or game about the content we learned in the previous lesson.” (T20) “I used a lot of quizzes and games. Before the holidays, I designed a Kahoot quiz with the context of the holiday and its traditions, and we played it in the class.” (T5)
Peer teaching in videoconference class sessions	4, 1%	“One hour each day was dedicated to peer teaching. The children taught each other how to bake, cook, do sports, etc. It was exciting to see how the other learners were listening to instructions from their peers, asking questions, and thanking them for the lesson.” (T74) “A student presented the artifact she designed to the entire class through a presentation and video.” (T10)
Self-directed teacher-led learning in small groups through videoconferencing	13, 3%	“The class was divided into four groups of students according to their levels and the willingness of the children for self-directed distance learning. Each group was assigned a fixed hour three times a week for self-directed practice.” (T20) “Apart from the Zoom sessions for the whole class I held meetings with smaller groups of students who attended the sessions. I met with them in groups of up to four students.” (T9)
Whole class videoconferencing for social cohesion	75, 19%	“We held social sessions in Zoom. We talked about how students felt, what they liked to do at home during the lockdown, and what place they liked to be in at home. This is the activity that most students participated in.” (T68) “We talked about their moods and we created ‘my personal box of powers’. We searched for positive implications of COVID-19, such as connecting the family, improving the environment, and less air pollution. We celebrated holidays together and celebrated a birthday for one of the children.” (T9)
Whole stratum/school activities and activities with family members	7, 2%	“Every week we initiated an online game for all the students in the school. Some families participated together with the students … we created escape room activities for the whole school community.” (T24) “Every morning we held social sessions via Zoom. We used Google form questionnaires and created whole stratum sessions to help learners solve math problems and answer their questions.” (T38)
Open discussions in synchronous or asynchronous mode	7, 2%	“In the class sessions I showed a short story in YouTube, and then we had a discussion about the subject of the story according to my guided questions.” (T62) “I sent questions to the students and opened forums in order to discuss these questions.” (T36)
Guidelines for individual asynchronous learning	88, 23%	“After the session I called those students who did not participate in the online class and asked if they needed any help.” (T35) “I recorded myself on WhatsApp every few days - explaining, demonstrating, reading, and giving assignments.” (T9)
Independent artifact design	26, 7%	“In my literature lesson I added a large collection of poems to our Google classroom - they are inspirational, optimistic, and empowering. Each student created an artifact based on the poem he chose and presented his work with an important quote from the poem. The artifacts were collected in a joint pool that was accessible to the wide audience.” (T3) “The task was to send pictures that documented artifacts that students prepared at home.” (T5)
Asynchronous collaborative learning	20, 5%	“Students had to choose a topic for a personal project. Each one posted his/her topic on a joint digital board. Students helped each other expand their topics and raised possible research questions.” (T65) “We prepared a dessert competition. The students wrote a recipe and through that we learned how to make an instructional text. We built a collaborative presentation and uploaded the recipes and pictures. We held a contest among the students and the winning dessert was chosen. At the end we had a joint recipe booklet.” (T6)
Practice through asynchronous tasks	12, 3%	“I created a game and quiz that helped my students practice.” (T20) “As a math teacher I gave my students short independent assignments to practice. In order to do the tasks, they could use video tutorials on the web.” (T2)
Assessment methods: Teacher feedbackKnowledge test	10, 3%	“I sent explanations to my students in a video I created. Then they had to perform exercises. Further, they were asked to scan or take pictures of their answers and send them back to me for feedback.” (T20) “After a virtual lesson using the Zoom platform, I made a short test for the students using a Google form.” (T124)

The table above shows that teachers integrated synchronous, asynchronous, and blended learning. This combination led to the implementation of a wide range of teaching and learning strategies. For example, many teachers used whole class teaching in their distance learning sessions. Some of these sessions were used to teach the usual subject matter, but many of the sessions aimed to maintain personal and social connections with the teacher and among peers, as well as to reduce the anxiety and stress of students. It was surprising and promising to find that teachers conducted individual and small group learning sessions in order to maintain teacher-student relationships and to assist students as needed. Joint school activities were also reported, some of which included family members. Other teachers chose an asynchronous approach and gave their students' guidelines for self-assignment tasks . Such tasks required students to function as independent learners and to manage their learning differently from what they were accustomed. Moreover, teaching strategies, as shown in Table 3, showed wide use of collaborative learning practices by students at home as well as during online sessions. In addition, part of the statements referred to how teachers monitored and assessed student performance on assigned tasks. Finally, we were surprised to find that very few teachers perceived the content broadcasted by the MoE as a useful resource in their online teaching.

## Discussion

As the COVID-19 pandemic has spread, online distance learning has become the main teaching method used worldwide (Huang et al., 2020; Kong, 2020; Schwartz et al., 2020). First, this section discusses the main pedagogical, technological, and organizational challenges and benefits of emergency remote teaching during COVID-19. Following that, we address pedagogical strategies that teachers employed for ERT.

### Challenges of Teachers and Students in Emergency Remote Teaching

The **first research question** referred to the main challenges and benefits of emergency remote teaching during COVID-19. Previous studies focusing on distance learning (e.g., Cheng, 2020; Hodges et al., 2020) presented the challenges of teachers in conducting effective use of distance learning in general, and ERT in particular. Similarly, our findings indicate various pedagogical, technological, systemic organizational, and personal challenges that teachers dealt with.

***The pedagogical* challenges** developed from the immediate necessity to move from traditional face-to-face classroom to distance learning. Challenges reported by teachers were based on the need to adapt their teaching methods to the new medium and the level of students. They needed to combine synchronous and asynchronous activities wisely in a blended learning mode. Teachers were also challenged to maintain ongoing personal contact with their students, facilitate their learning, and meet their emotional needs during that time. As to the related challenges of students, teachers emphasized the difficulties of students to function as independent learners and develop their self-regulated skills. They were required as well to collaborate and to develop a routine for continuous learning. Teachers provided ongoing scaffolding and assistance to develop self-regulation skills. However, students were challenged by the need to set learning goals, determine their progress, choose learning techniques, manage emotions and behavior during learning processes, and conduct self-assessments (Johnson & Davies, 2014).

Interestingly, students' emotional distress was rarely mentioned as a challenge during this period. It is possible that a variety of social activities conducted by the teachers helped reduce anxiety, and therefore a feeling of distress was not prevalent in the data. In a similar vein, previous studies on education in times of crisis (Burde et al., 2017) showed that social activities contributed to students' sense of security. Providing children in ERT situations with structured, meaningful, and creative activities in a school setting or in informal learning spaces, improves their emotional well-being and has positive implications on their behavior.

**The *technological challenges*** of teachers emerged from the need for immediate use of various digital tools and LMS platforms, often with insufficient training. In addition, the data shows that teachers lacked appropriate equipment to conduct distance learning. Vlachopoulos (2020) addressed the issue of equipping teachers in order to facilitate their optimal work from home. The current study shows that in some homes one computer had to meet the needs of both the teacher and his/her children as students. This is a significant technological challenge for which solutions must be found at the national system level (Sälzer & Roczen, 2018).

**The *systemic organizational challenges*** reported by our participants indicated a lack of coordination between the national education system and the local school systems. These included negative attitudes of teachers and a sense of lack of support from the MoE. Conflicting instructions caused confusion and difficulties in schools' internal organization in moving quickly to ERT. *On the national level*, the MoE is required to support and guide schools, provide them with digital content, platforms, and tools, and train teachers (Razak et al., 2019). Their support and guidance are especially important in times of crisis (Cheng, 2020; Huang et al., 2020), and without a well-designed guidance protocol schools will find it difficult to implement ERT activities (Xie & Yang, 2020). According to our findings, lack of support on the national level led to a lack of cooperation from parents and to criticism expressed in the media that distance online learning is not as effective as face-to-face instruction. Although for one week each year the Israeli MoE conducts an emergency learning exercise for all educational institutions (MoE, 2020), this training does not seem to be sufficient for preparing teachers for the challenges of real ERT. Most of our participants recognized the importance of this exercise for helping them in ERT design but argued that practicing one week per year is not enough. Similarly, research literature shows that many teachers and students did not receive adequate training for distance teaching and learning, and therefore were unprepared for the real emergency event (Burde et al., 2017; Hodges et al., 2020).

*At the school level*, teachers presented challenges that needed to be resolved, such as adapting a school schedule for online learning sessions. A lack of planning created overlapping synchronous lessons targeted for the same students and an overload of assignments. Accordingly, schools had to organize a systemic solution, for example, through micro-learning matrix (see: Shamir-Inbal & Blau, 2020), in order to enable students of all age levels to learn different subjects at diverse hours throughout the day and to conduct various synchronous and asynchronous activities.

**The *personal challenges*** addressed the participants' difficulties in coping with home and family restrictions resulting from the COVID-19 pandemic. Namely, when teachers worked, their own children were also at home and needed their attention and help as parents for their learning processes. These challenges concerning teachers' personal and family constraints and work-life balance are consistent with literature (Froese-Germain, 2014; Weisberger et al., 2019). Accordingly, boundaries between working time, family time, and leisure time are important elements in an individual’s life, which need to be separate. Thus, during COVID-19 teachers felt torn between their duties at home and their duties as teachers.

### Benefits of Teachers and Students in Emergency Remote Teaching

Although distance learning was perceived as a challenging task for teachers and students, most of the teachers who participated in this study embraced ERT as an empowering event and an opportunity for personal and professional growth. Moreover, for many educational practitioners and researchers, the COVID-19 crisis has been considered a unique opportunity that can support both students and teachers in bridging the gap left by conventional (face-to-face) education and promote adoption of more appropriate pedagogical methods (Vlachopoulos, 2020).

***On the pedagogical level***, according to the findings, teachers acquired new teaching and technological skills by adapting various distance learning strategies. They were able to maintain a learning routine in various subject matter and age groups. They were also able to design different types of teaching activities suitable to the pandemic reality. The ability to develop digital content affects teachers’ perceptions of TPACK (Koh et al., 2015). A previous study (Blau & Shamir-Inbal, 2017a) showed that teachers prefer to use existing learning activities, which were made accessible by the MoE, and are less likely to be engaged in instructional design. However, it emerged from our data that during ERT teachers preferred to design their own learning activities and free themselves from the routine content. This is consistent with previous studies highlighting the importance of designing and tailoring teaching activities by teachers for their students (McKenney et al., 2015; Shamir-Inbal et al., 2009). Moreover, teachers who are more open to designing and adapting existing digital content become more professional in their teaching (Koh et al., 2015).

This experience of pedagogical design contributed to teachers' ***personal empowerment*.** Participants reported that ERT became an opportunity to think and act outside the box and to develop different, unconventional, and creative learning methods. They felt a sense of accomplishment and professional success based on the students' well-being and their ongoing communication with them. This experience of ERT further emphasized the importance of giving a free hand to personal initiatives (Koh et al., 2015; McKenney et al., 2015). The sense of empowerment and success reported by teachers was based on their involvement in the ERT challenges and their ability to overcome them.

As the teachers adapted their role to ERT, they assisted **students** in dealing with the challenge of becoming self-regulated learners. Studies have shown that students’ autonomous learning can improve learning performance and promote the cultivation of lifelong learning skills (Shamir-Inbal & Blau, 2020; Xie & Yang, 2020). Teachers perceive self-regulated learning skills as a coping strategy of lifelong learners that usually is not required in the classroom (Hadad, Shamir-Inbal, Blau & Leykin, 2020; Shamir-Inbal & Blau, 2020), and ERT was an opportunity to promote their development (Kong, 2020). However, the data regarding students needs to be treated with caution, since it is based on reports of teachers and was not triangulated with students' own perspectives.

***Technology*** played a key role in the ERT experience. Many of our participants reported that they were introduced to a range of technological tools which they needed to master. These technological tools enabled integrating up-to-date pedagogical methods, monitoring student performance, and maintaining ongoing e-communication between teachers, students, and their parents. These implications of technology integration are beneficial in both ERT and offline learning in the classroom (Blau & Hameiri, 2017; Engelbertink et al., 2020).

***On the systemic organizational level***, the lack of consistent national guidelines led to various bottom-up initiatives. Principals and teachers on the school level were free to design tailored teaching activities and be creative. Teachers reported a high level of responsibility for dealing with ERT through mutual support and exchanging learning activities they designed or adapted. In addition, they reported mutual help in mastering required digital tools, as well as improved intra-school coordination which developed over time. This coordination and mutual support made it possible to conduct successful ERT and strengthen digital school culture. Maintaining communication, collaboration, and mutual support within the staff enables better coping with challenges arising in technology-enhanced teaching and learning (Blau & Shamir-Inbal, 2017a; Shamir-Inbal et al., 2009), especially during ERT (Hodges et al., 2020). We believe that the new school culture that has emerged during ERT can foster school leadership and promote the integration of innovative technologies into the school curriculum and daily classroom activities (Blau & Presser, 2013; McKenney et al., 2015; Razak et al., 2019; Shamir-Inbal et al., 2009).

An additional factor on the systemic level is **parental involvement and support** for student learning during emergencies and in maintaining learning routines and ongoing communication (Xie & Yang, 2020). According to teachers, parental involvement was problematic at the beginning of the crisis, but this changed as teachers and families adapted to the new reality. Parents became more involved and supportive of the teachers and of schools in general. This may lead to a change in understanding the importance of the teacher's role in children's daily routines during the lockdown. An additional change may be a strengthening of teacher status (Vlachopoulos, 2020), as well as an awareness of the importance of parental cooperation with teachers on a regular basis (Blau & Hameiri, 2017). Note that parental involvement and support for students' learning during the pandemic was also based on teacher reports. Future studies need to triangulate teachers' perspectives with the reports of parents and students.

### Pedagogical Strategies for Emergency Remote Teaching

Effective use of technology requires adapting pedagogical methods to innovative opportunities (Xie & Yang, 2020; Yen, 2020). The findings revealed a variety of teaching strategies used during ERT. These strategies were used in synchronous sessions, asynchronous tasks, and in blended learning which combined these two teaching-learning modes.

During **synchronous sessions** teachers could hold class meetings for teaching-learning purposes in their subject-matter, for e-communication with students, and for enhancing social cohesion. Synchronous lessons were held via videoconferencing tools, such as *Zoom* or *Meet* applications, that allowed two-way communication during the lessons (Weiser et al., 2018). The use of synchronous *whole class learning* sessions took place at different stages of the instruction and for different pedagogical needs. These sessions were used for presentations, demonstrations, and providing explanations of learning tasks in whole class teaching. In some cases, they expanded to include participation of family members, for example, to perform joint digital game activities. Moreover, the whole class sessions were used for social purposes to keep in touch during ERT. In these sessions, students could share their feelings and activities at home. The main purpose of this kind of meeting was an emotional release for students and to identify severe distress that might require further counseling. These sessions strengthened students' bond with their teacher and social cohesion with the class (Laprairie & Hinson, 2006; Rice, 2006). Learning in *small groups* was employed to provide guidance and individual support in accordance with various student needs. Such guidance is important for promoting students' self-regulation skills and enabling them to continue acting as independent learners (Blau & Shamir-Inbal, 2018). In a few cases, learning in small groups was integrated into whole class sessions; for example, after initial explanations students were split up to *Zoom* Breakout Rooms to conduct learning tasks and, towards the end of the session, returned to the plenary and reported their outcomes to the class.

Despite its benefits, synchronous learning is not sufficient to create active learning and learner involvement over time (Blau & Shamir-Inbal, 2017b; Ouyang et al., 2020; Weiser et al., 2018). The advantage of **asynchronous learning** is that it is carried out on a flexible schedule. It allows learners to manage their learning independently, conduct deeper discussions, be active in practicing and creating artifacts, and collaborate with their classmates (Blau & Shamir-Inbal, 2018; Ouyang et al., 2020). In addition, it enables teachers to guide learning through structured and detailed scaffolding in order to help learners work independently (Romero-Hall & Vicentini, 2017; Yen, 2020). According to this study, asynchronous learning was also performed at different stages of the instruction, in different formations, and for different pedagogical needs. Asynchronous tasks involve independent learning activities, such as practice, participation in offline discussions, active information processing, or artifact creation. These can be conducted independently or in groups, using shared documents and/or online applications (Blau, Shamir-Inbal, & Avdiel, 2020). Such asynchronous tasks can be accessible to learners as prompting tasks which enhance curiosity and arouse students' prior knowledge or for summarizing a topic. Moreover, this independent learning enables teachers to guide and support their students during the entire learning process in order to improve it.

It is important to note that 46% of the teachers participating in this study reported that they conducted blended learning, combining synchronous and asynchronous communication with their students. This combination profits from advantages of each learning mode (Blau & Shamir-Inbal, 2017b), can empower instruction, and is an important current challenge that educational systems need to face (Romero-Hall & Vicentini, 2017). In order to promote effective blended ERT, suitable professional development (TPD) courses which combine synchronous and asynchronous activities are needed. Such courses enable in-service teachers to experience blended learning as trainees and afterwards to integrate it in their teaching (Desjardins & Bullock, 2019; Hadad, Shamir-Inbal, & Blau, 2020; Ndongfack, 2015; Shamir-Inbal & Blau, 2020). Figure 2 summarizes synchronous and asynchronous characteristics and pedagogical strategies found in this study.

As Figure 2 shows**, *synchronous learning*** is characterized by immediate interpersonal communication in whole class or entire stratum settings. Such settings enable discussions of learning content or social issues, as well as demonstrations and presentations by teachers or students. ***Asynchronous learning*** is characterized by flexibility in time and place and requires self-regulated learning skills. It enables learning at a personal pace and on different levels, as well as development of digital literacy skills through individual or collaborative digital tasks. ***Blended learning*** seems to be the optimal ERT mode. Knowledge acquisition and information sharing are performed in a synchronous mode, while other aspects of personal, independent, and collaborative learning are performed asynchronously. This blended learning mode allows teachers to employ a variety of strategies according to different pedagogical needs and steps of the learning process (Desjardins & Bullock, 2019). The findings suggest that the ability of teachers to conduct blended ERT adapted to the needs of their students created a sense of empowerment. This might explain the choice of our participants to design ERT activities by themselves instead of using existing digital content accessible to their schools.

Unfortunately, this study shows that only a small number of teachers addressed **assessment methods** as part of the teaching-learning process. Embedded assessment is a fundamental aspect of instruction that helps students improve their work and contains special challenges and affordances when conducted in online environments (Blau & Shamir-Inbal, 2018; Kearns, 2012). Therefore, the issue of planning embedded assessment through routine and ERT should be included in teacher training in order to help teachers expand their experience in using diverse assessment methods at different stages in their pedagogical design (Hadad, Shamir-Inbal, Blau, & Leykin, 2020).

## Conclusions, Implications, and Future Directions

This study describes teacher experience in leading ERT in K-12 using synchronous, asynchronous, and blended instruction. Although this study was conducted in a specific Israeli context, the exploration of benefits and challenges of ERT and pedagogical strategies during times of crisis such as the COVID-19 pandemic are relevant to the global research and education communities. The research presented valuable data for instructors engaged in ERT and can assist in designing suitable blended learning. Based on the study findings regarding pedagogical strategies summarized in Figure 2, we recommend combining both synchronous and asynchronous learning in ERT. Synchronous learning is characterized by immediate assistance and interpersonal communication with a teacher and peers (Weiser et al., 2018). Asynchronous learning is characterized by flexibility in time and space that enables preparing complex, authentic, and creative learning outcomes (Shamir-Inbal & Blau, 2021).

Further, this study raises the need to continue experiencing distance learning on a regular basis as part of the school agenda. Incorporating blended learning in school practices on a regular basis may strengthen both pedagogical strategies of digital learning and the self-regulated learning and teamwork skills of students (Blau & Shamir-Inbal, 2018; Kong, 2020). These skills were found to need improvement in the current study. In this way we will be able to convert a lemon into lemonade, so that ERT becomes an opportunity to be a blessing rather than a curse.

The main limitation of this study is its self-report methodology. Future research should include observations of teacher behavior in ERT and an analysis of online activities conducted during this period. In addition, this study was conducted after a two-month period of ERT. Future directions need to include longitudinal studies that explore the development of pedagogical strategies during ERT. Moreover, it is important to understand the implication of ERT experience on post-COVID-19 technology-enhanced, face-to-face, and blended teaching and learning combining online and offline interactions. Finally, future research needs to search for effective assessment techniques for online learning.
